# Social Anxiety, Tremor Severity, and Tremor Disability: A Search for Clinically Relevant Measures

**DOI:** 10.1155/2013/257459

**Published:** 2013-07-31

**Authors:** Duane A. Lundervold, Patrick A. Ament, Peter Holt

**Affiliations:** ^1^Saint Luke's Hospital, Kansas City, MO, USA; ^2^Plaza Primary Care and Geriatrics, 4440 Broadway, Kansas City, MO 64111, USA; ^3^University of Central Missouri, USA

## Abstract

*Background*. While social anxiety has been reported among essential tremor (ET) patients, very little is known about the relation between self-report measures of social anxiety, tremor severity and disability, and cognition. *Methods*. Sixty-three individuals diagnosed with ET took part in a comprehensive study examining neurocognition and behavioral functioning. A psychiatric diagnostic interview, three social anxiety questionnaires, and an idiographic-based behavioral assessment to pinpoint anxiety provoking situations and related distress were completed. *Results*. Thirty percent of the participants met diagnostic criteria for social anxiety disorder (SAD). Social anxiety questionnaires were negligibly related to tremor severity and disability. Idiographic behavioral assessment of subjective distress was moderately related to resting tremor severity and disability and strongly related to social anxiety questionnaires scores. Only one cognitive variable was related to tremor severity. *Conclusions*. These findings suggest that (a) self-report measures of social anxiety with ET patients may underestimate distress; (b) emphasis on tremor severity may be misleading; (c) tremor disability may be a more sensitive and functional measure related to cognition and effect; (d) SAD is wide spread and does not appear to be related to dysregulated executive function; and (e) development of an ET-specific measure of social anxiety is called for.

## 1. Introduction

Over the last 25 years, there has been a gradual acknowledgment that essential tremor (ET) is comprised of much more than a benign rhythmic oscillation of a limb [1–4]. A significant amount of data has been accumulated indicating ET is a multifaceted syndrome with multiple neurologic causal pathways, and, possibly, is a family of diseases [5]. Concurrent with closer examination of neurologic connections has been an increasing awareness of the breadth and magnitude of nonmotor symptoms, including cognition, disability and well-being [3, 4]. Evidence is emerging that ET is related to subtle, but significant cognitive impairment; however, the relationship between changes in cognition and nonmotor features of ET have rarely been examined [2, 3]. Data supporting impaired cognition is primarily based on clinic samples or individuals being considered for deep brain stimulation implant surgery. Such samples are not representative the population of individuals with ET and those patients who present for consideration of implant surgery are the most severe cases. Little is known about independent living, community dwelling, non-clinic based ET samples in terms of cognition, disability and well-being.

 Busenbark et al. [1] and Metzer [6] were the first to observe that despite pharmacotherapy large numbers of patients with ET reported significant embarrassment and diminished quality of life in the psychosocial domain. While significant, embarrassment is likely to be too narrow a description of the behavior evoked by situations patients with ET encounter. ET patients report far more than “embarrassment” which can too easily be trivialized by health care providers and others. Embarrassment, an emotional response, is a piece of social phobia or social anxiety disorder (SAD) [7]. The Diagnostic and Statistical Manual (DSM) [7] exclusionary criteria applied in the diagnosis of psychiatric disorders may have limited examination of the extent and characteristics of social anxiety that frequently co-occurs with ET. Employment of this criterion restricts the diagnosis of SAD to cases where the anxiety is unrelated to a medical disorder such that a “pure” anxiety disorder may be detected. With respect to ET, doing so limits our understanding of the relation between anxiety and medical disorders and whether anxiety is a precursor to a medical disorder, a pathological consequence, or a conditioned emotional response brought about through learning as proposed by Lundervold and Poppen [8].

 Reports of SAD among movement disorder clinic samples range from 33–42% [9, 10]. SAD is comprised of three response components: (a) emotion (fear and anxiety and embarrassment); (b) cognition (worry and anxious anticipation), and (c) escape and avoidance behavior. Solely focusing on embarrassment ignores the other two vitally important response components leading to insensitive assessment of the quality and characteristics of SAD is likely to lead to poorer treatment outcomes and limited use evidence-based treatments that address avoidance behavior [11]. Accurate behavioral assessment requires that instruments are valid and reliable in order to prevent measurement error. For example, Schneier et al. [9] reported that ET patients experienced greater anxiety in performance versus social interaction situations. Topçuoğlu et al. [10] reported increased avoidance behavior among ET-SAD compared to non-ET SAD patients. These patients also scored higher on performance-related fear. Tremor severity was not related to measures of SAD, while the level of social anxiety was found to be directly related to increased disability and decreased quality of life. These findings point to the important and unique situational components that evoke social anxiety and the multiple modes of responding that comprise all anxiety disorders. The phenomenology of SAD among ET patients may be different and points to the need to examine the utility of current measures of SAD with this same group. Moreover, there is a clear need to establish reliability and validity measures of social anxiety used with patients with ET. Identification of such measures would lead to more precise assessment of social anxiety and evaluation of treatment outcome of SAD among patients with ET. The purpose of the current study was to examine the relation between (a) self-report measures of social anxiety, tremor disability, and clinical ratings of tremor severity and (b) measures of cognition and anxiety. 

## 2. Method

### 2.1. Participants

Sixty-three community dwelling individuals (MN age 67.13 years; SD 14.02) diagnosed with ET took part in a comprehensive study examining neurocognition and behavioral functioning. The medical examination was performed by the participant's primary care physician or movement disorder specialist who then provided the diagnosis. All the participants were Caucasian, reasonably well educated (MN14.82 years, SD 2.77), and with close to equal representation based on sex (46% male). Mean duration of ET based on diagnosis was 15.87 years (SD 11.49). Mean income was $61,115 (SD 7,351). Nearly 70% of the sample was retired; approximately 21% were working full or part time. Slightly more than 21% of the participants reported an immediate family history of ET, 55% reported that alcohol altered tremor severity, though 33% reported abstaining from alcohol. Finally, 60% of the participants were on ET-related medication, and 38% were prescribed anxiolytic medication. 

 Participants were recruited through direct and email contact and a research announcement posted on the International Essential Tremor Foundation website. The announcement asked for persons with a diagnosis of ET to take part in a study of ET cognition and anxiety. Any person with a diagnosis of ET could take part. Data were collected in an office suite within a primary care medicine building. The research was approved by the saint Luke's Hospital Institutional Review Board, and informed consents was obtained from participants. 

### 2.2. Measures

*Psychiatric Diagnostic Interview-Revised (PDI-R). *The PDI-R is a structured diagnostic interview that has demonstrated to have good reliability and validity [12]. The PDI was completed by the first author.

*Clinical Rating of Tremor Severity (CRTS).* The magnitude of tremor severity was assessed using a nine-point rating system that we have used in our earlier research [13–15]. Good inter rater agreement and positive correlations between severity ratings and motor task performance have been obtained. Tremor assessment was conducted by the first author.

*Tremor Disability Scale-Revised (TREDS-R)*. The TREDS-R, based on the original Bain et al. instrument [14, 16], is comprised of 20 items that measure the degree of disability in performance of activities of daily living related performance of the hands. TREDS-R is a valid and reliable measure of tremor disability with high internal consistency.

*Anxiety Questionnaires. *Three validated self-report measures of social anxiety were administered. The Social Phobia Inventory (SPIN) [17] is a 17-item instrument designed to assess fear, avoidance, and physiological dimensions of social anxiety. Good test-retest reliability (*r* = .78), internal consistency (*r* = .56–.76, depending on subscale) and convergent validity based on interview (*r* = .57–.66) have been reported. The Social Interaction Anxiety Scale (SIAS) and Social Phobia Scale (SPS) developed by Mattick and Clarke [18] also were used. The SIAS assesses anxiety during verbal interactions, whereas the SPS assesses anxiety during performance of tasks while being observed. Good internal consistency and test-retest reliability have been demonstrated [19, 20].

*Subjective Unit of Discomfort (SUD). *SUD ratings, widely used in cognitive behavior therapy and behavior therapy outcome research, provide an idiographic assessment of discomfort related to specific fear eliciting situations [21]. A 10-point SUD rating scale was used to measure the extent of discomfort in anxiety provoking situations. An SUD score was obtained only if the participant reported a distress evoking situation. Higher scores indicated greater distress.

*Measures of Cognition. *Immediate and delayed recalls, perception, and language domains were assessed. Immediate and delayed recalls of a word list were assessed using the procedure developed by Kalbe et al. [22]. Perception was assessed using two separate visual discrimination tasks. To assess language functions, the phonemic verbal fluency task (F-A-S) [23] was used, whereas categorical fluency was examined using animals, fruits, and vegetables categories [24]. In each case, the participant was instructed in the task and given 60 seconds to generate as many responses as possible.

### 2.3. Design and Analysis

Data from all individuals taking part in the research were analyzed. Self-report questionnaires demonstrated nonlinearity between variables, and, as a result, a Spearman rho correlation analysis was used. Measures of cognition, tremor severity, TREDS-R, and SUD ratings were linearly related, and a Pearson correlation was used.

### 2.4. Procedure

Participants completed cognitive and behavioral measures in a randomized order. For ease of administration, social anxiety questionnaire items were read to the participant who then provided a rating for each question using a visual aid which displayed the rating scale employed for that questionnaire. Idiographic behavioral assessment of anxiety provoking situations was conducted by asking the participant to describe any situation where anxiety and distress occurred, including situations directly related to tremor. The description was manually recorded by the researcher who then asked the participant to provide an SUD rating for that situation. A visual aid was provided to assist the participant in making the rating. The participant was then asked if there were any other situations that evoked anxiety, and the process was repeated as before.

## 3. Results

Thirty percent of the sample met diagnostic criteria for social phobia based on the PDI-R psychiatric interview conducted by the first author (D. A. Londervold). The DSM-IV exclusionary rule was not applied in making the diagnosis.  Table 1 displays mean, standard deviation and minimum and maximum scores for measures of cognition, tremor severity, and disability. The relatively low level of tremor severity with kinetic being more severe than postural tremor is of interest. Tremor disability, as measured by the TREDS-R, indicated clinically significant impairment (+1.5 SD) in motor performance and resultant disability compared to normative data [16, 25].

Nearly 78% of the sample reported distress in at least on situation and 33% reported distress, based on SUD ratings, in two or more situations. To calculate the mean SUD scores obtained from only those participants providing ratings were used. If a participant provided an SUD rating for more than one situation the mean score was calculated for that individual. A mean SUD rating of 6.52 (SD 1.71) was obtained indicating a moderate degree of distress (see Table 2). Group mean scores for the SPIN, SPS, and SIAS suggest minimal social anxiety, based on empirically derived cutoff scores as the criterion, with the exception of the SPS. Significant variability in self-reported social anxiety was observed. Based on cutoff scores, 22% were above the clinical cutoff for the SIAS, 41% for the SPIN, and 39% for the SPS. 

SUD ratings were moderately related to the SPIN, SPS, and SIAS (*r* = .32–.38, *P* ≤ .05) with the SPS most strongly related to SUD ratings (*r* = .38, *P* = .008). SUD ratings were associated with right resting (*r* = .33, *P* = .02) and left kinetic (*r* = .29, *P* = .04) tremor severity ratings. SIAS, SPS, and SPIN were consistently related to right and left resting tremors (*r* = .31–.41, *P* = .01–.005); however, only SUD ratings and the SPS were related to the TREDS-R (*r* = .40, *P* = .001–.004). TREDS-R was associated with line orientation scores (*r* = − .26, *P* = .04) and approached significance for facial recognition (corrected score) (*r* = − .25, *P* = .053). The inverse relation between right and left kinetic tremors and delayed recall approached significance (*r* = − .20–.24, *P* = .06–.12). No other relationships were consistently observed.

## 4. Discussion

 Earlier research with clinic-based and epidemiological samples of patients with ET has found subtle but important change in cognition. Other research with clinic-based samples has reported high levels of SAD, with increased anxiety in performance relative to social interaction domains. Our research is the first to comprehensively assess and report findings examining the relationship between social anxiety, tremor severity and disability, and cognition. Consistent with past reports, SAD is a common occurrence among community dwelling individuals with ET. The 30% prevalence of SAD in our sample is consistent with that reported among clinic-based populations who are likely to have more severe tremor suggesting that tremor severity is not the driving force behind SAD. The high prevalence of SAD and the correspondingly high use of anxiolytic medication within our sample suggest that pharmacotherapy is the primary means of treatment for SAD among this ET sample; however, anxiolytic medication is also used to treat tremor itself. It is unclear if anxiolytic medication was prescribed for tremor per se or as a means to dampen arousal in anxiety provoking situations. It may also be that the participants prescribed anxiolytic medication were not receiving optimal treatment.

 Despite the use of anxiolytic medication for SAD, significant distress was reported based on the self-report questionnaires and idiographic behavioral assessment results. Such medication may also result in iatrogenic effects that affect cognition and motor performance. These results point to the weak positive effect of anxiolytic medication alone in the treatment of ET-SAD. The effectiveness of cognitive behavior therapy, shown to improve SAD among populations, needs to be examined in the context of ET-SAD.

 The lack of meaningful relationships among self-report questionnaires (SIAS, SPS, and SPIN), tremor severity and disability is an important finding for several reasons. First, from a purely measurement perspective, the SPIN, SIAS SPS are likely to underestimate the magnitude of distress because they are not assessing the conditions under which patients with ET report social anxiety, which are primarily performance-based situations. Use of a control group and an ET specific measure of social anxiety would allow a direct comparison and allow testing of this hypothesis. Secondly, the SPIN and SIAS were only related to resting tremor, which was extremely mild (MN < 1.00) and unrelated to tremor disability. This is a puzzling finding, especially if tremor severity produces disability and severity is the causal link in producing social anxiety. It may be that some of the sample participants may have had mild symptoms of Parkinson's disease and these symptoms went undetected by the participant's medical provider. Alternatively, resting tremor in ET patients is not uncommon, with a prevalence rate of 20% reported [26]. Given the extent of social anxiety within the ET patients, it is also likely that resting tremor may have been due to anxiety itself, as tremor is a symptom of social anxiety. In sum, the self-report questionnaires examined are likely to provide unreliable and invalid estimates of treatment outcome relative to ET-SAD. 

In contrast to prior research on cognition among ET patients, we found few consistent relationships between multiple measures of cognition, anxiety, tremor severity, or tremor disability. Two visual perceptual tasks were related to the TREDS-R suggesting that, among this nonclinic sample of ET participants, subtle but meaningful impairment in perception was observed. Evidence suggests that both tasks assess right hemisphere integrity and do not rely on memory. Another intriguing finding is an inverse relation between kinetic tremor severity and delayed recall that approached significance. This result lends tentative support to earlier observations regarding ET and dementia [27]. Replication of this inverse relation is needed using the same measures and a larger nonclinic sample. The limited number of significant relations between cognition, tremor severity and disability may be due to sample characteristics, mild degree of tremor severity among the participants, or measurement procedures. The lack of inter rateragreement for tremor severity could be a factor contributing to the lack of relationship between tremor severity and cognition. Cognitive measures were selected based on past ET research using the same instruments and reporting change in cognition. Administration of measures of cognition is standardized thus limiting measurement error. The sample recruited for the research was independent living, community dwelling adults in contrast to patients attending a movement disorder clinic or nursing home residents, although they were not randomly selected as in epidemiological studies of ET. The nine-point Bain rating scale may have affected the results, too, by providing three grades of severity with three levels within each gradation. Finally, the weak relationship between measures of cognition and social anxiety suggests that anxiety and SAD may not be due to neurodegenerative processes or dysregulation of executive function, but rather anxiety is a conditioned emotional response brought about through operant and respondent conditioning processes [8]. Further research using a broader array of measures of executive functioning and cognition is needed to sort this out. Further research is needed examining the prevalence and characteristics of social anxiety among movement disordered patients. Finally, patients with ET-SAD may benefit from treatment procedures based on principles of behavior and conditioning [8]. 

## Figures and Tables

**Table 1:** Descriptive statistics for measures of cognition, tremor severity, and tremor disability.

	*N*	Minimum	Maximum	Mean	Standard deviation
BFR (adjusted)^1^	63	24.00	55.00	44.89	5.73
Categorical fluency^2^	63	14.00	66.00	39.41	11.22
Delayed recall^3^	63	1.00	10.00	5.17	1.87
FAS perseveration	63	.00	5.00	.95	1.30
FAS total^4^	63	11.00	60.00	34.52	11.74
Immediate recall	63	7.00	18.00	12.40	2.32
JOL (adjusted)^5^	63	14.00	52.00	27.63	4.92
Left kinetic	63	.00	8.00	2.94	1.89
Left postural	63	.00	8.00	1.94	1.97
Left resting	63	.00	5.00	.56	.88
Right kinetic	63	.00	7.00	2.83	1.85
Right postural	63	.00	8.00	1.94	1.90
Right resting	63	.00	5.00	.81	1.00
TREDS-R^6^	63	20.00	54.00	33.41	8.48

^1^Benton facial recognition test of perception. Individual scores were adjusted using age, education, and sex corrections variables.

^2^Individual categorical fluency scores must be interpreted using age, education, and sex corrections variables.

^3^Delayed recall scores <4 indicates impaired performance according to Kalbe [22].

^4^Individual FAS scores must be interpreted using age, education, and sex corrections variables.

^5^Benton judgment of line orientation test of perception. Individual scores were adjusted using age, education, and sex corrections variables.

^6^Mean score for the TREDS-R is 20 based on a nonmovement disordered sample according to Lundervold [25].

**Table 2:** Descriptive statistics for SUD ratings and social anxiety questionnaires.

	*N*	Minimum	Maximum	Mean	Standard deviation
SUD	49	2.00	9.75	6.52	1.71
SPIN	63	1.00	65.00	18.44	14.73
SPS	63	.00	77.00	25.81	18.06
SIAS	63	.00	64.00	21.75	16.51
